# Structural Basis for the Substrate Inhibition of Proline Utilization A by Proline

**DOI:** 10.3390/molecules23010032

**Published:** 2017-12-23

**Authors:** David A. Korasick, Travis A. Pemberton, Benjamin W. Arentson, Donald F. Becker, John J. Tanner

**Affiliations:** 1 Department of Biochemistry, University of Missouri, Columbia, MO 65211, USA; korasickd@missouri.edu; 2 Department of Chemistry, University of Missouri, Columbia, MO 65211, USA; tpemb@sas.upenn.edu; 3 Department of Biochemistry, Redox Biology Center, University of Nebraska, Lincoln, NE 68588, USA; ben.arentson@gmail.com (B.W.A.); dbecker3@unl.edu (D.F.B.)

**Keywords:** flavoenzyme, proline dehydrogenase, l-glutamate-γ-semialdehyde dehydrogenase, substrate inhibition, X-ray crystallography

## Abstract

Proline utilization A (PutA) is a bifunctional flavoenzyme that catalyzes the two-step oxidation of l-proline to l-glutamate using spatially separated proline dehydrogenase (PRODH) and l-glutamate-γ-semialdehyde dehydrogenase (GSALDH) active sites. Substrate inhibition of the coupled PRODH-GSALDH reaction by proline is a common kinetic feature of PutAs, yet the structural basis for this phenomenon remains unknown. To understand the mechanism of substrate inhibition, we determined the 2.15 Å resolution crystal structure of *Bradyrhizobium japonicum* PutA complexed with proline. Proline was discovered in five locations remote from the PRODH active site. Most notably, strong electron density indicated that proline bound tightly to the GSAL binding site of the GSALDH active site. The pose and interactions of proline bound in this site are remarkably similar to those of the natural aldehyde substrate, GSAL, implying that proline inhibits the GSALDH reaction of PutA. Kinetic measurements show that proline is a competitive inhibitor of the PutA GSALDH reaction. Together, the structural and kinetic data show that substrate inhibition of the PutA coupled reaction is due to proline binding in the GSAL site.

## 1. Introduction

**Figure 1:**
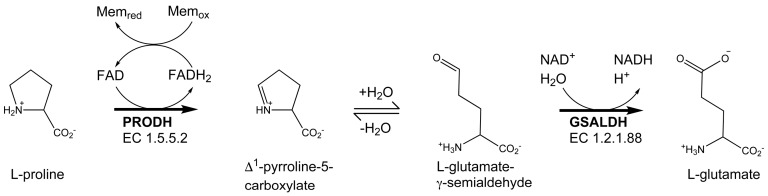
The reactions catalyzed by PutA.

Proline utilization A (PutA) proteins are bifunctional enzymes that catalyze the oxidation of l-proline to l-glutamate (Figure 1) [1,2]. PutAs contain two spatially separated active sites that catalyze the two reactions of proline catabolism. The proline dehydrogenase (PRODH) active site catalyzes the oxidation of proline to Δ^1^-pyrroline-5-carboxylate (P5C) with concomitant reduction of the flavin adenine dinucleotide (FAD) cofactor. The intermediate P5C is in equilibrium with its hydrolysis product, l-glutamate-γ-semialdehyde (GSAL). The latter species is the substrate for the GSAL dehydrogenase (GSALDH) active site of PutA, which catalyzes the nicotinamide adenine dinucleotide (NAD^+^)-dependent oxidation of GSAL to glutamate. The two active sites are separated by a linear distance of ~40 Å and connected by a tunnel through which the intermediate P5C/GSAL is transferred via substrate channeling.

The steady-state kinetic properties of the coupled PRODH-GSALDH reaction have been measured for three PutAs, and in all cases, substrate inhibition by Pro has been observed [3,4,5]. In the steady-state coupled assay, the enzyme is supplied with proline, NAD^+^, and an electron acceptor for the FAD coenzyme, and the production of NADH is monitored. The reaction rate displays non-Michaelis–Menten behavior in that, above a certain proline concentration, the rate decreases with increasing proline. This result is consistent with substrate inhibition, and fitting of the reaction velocity data to a substrate inhibition model has yielded *K*_i_ values in the range of 24–263 mM for the various PutAs examined. In particular, the enzyme used in the present work, PutA from *Bradyrhizobium japonicum* (BjPutA), has a *K*_i_ of 24 mM [3]. Although substrate inhibition in the coupled reaction appears to be a hallmark of PutA, the structural basis for this phenomenon has not been elucidated.

Herein we report the first structure of a PutA with proline bound in locations outside of the PRODH active site. The structure of BjPutA shows proline bound to the GSAL site of the GSALDH active site. The structure implies that inhibition of the GSALDH reaction by proline is the basis of substrate inhibition of the PutA coupled reaction. Kinetic measurements confirm that proline competitively inhibits the GSALDH activity of BjPutA. These results show that substrate inhibition of the PutA coupled reaction is due to proline binding in the GSAL site.

## 2. Results

### 2.1. Overall Structure

The BjPutA C792A variant was used for *X*-ray crystallography. Cys792 is the catalytic cysteine of the GSALDH active site, and C792A is devoid of GSALDH activity, but has wild-type PRODH activity [6]. Crystals of C792A are yellow, indicating that the FAD is oxidized. Soaking the crystals in 2 M proline bleached the yellow color, consistent with reduction of the FAD cofactor. After the bleaching was complete, the crystals were flash-cooled in liquid nitrogen to trap the reduced state of the enzyme. X-ray diffraction data were collected on a flash-cooled crystal, and the structure was determined at a 2.15 Å resolution (Table 1).

**Figure 2:**
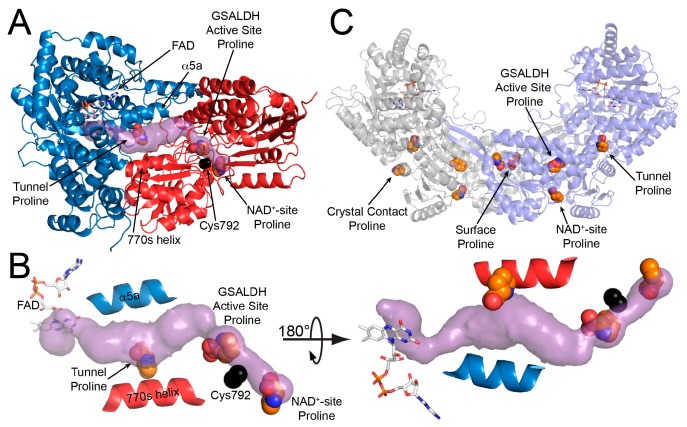
The crystal structure of BjPutA C792A. (**A**) The protomer of BjPutA C792A. The PRODH module is colored blue. The GSALDH module is colored red. The purple surface represents the substrate-channeling tunnel. For reference, the two α-helices that border the central section of the tunnel are noted (α5a, 770 s helix). (**B**) Close-up view of proline molecules bound in the GSALDH active site and the middle of the tunnel. (**C**) The dimer of BjPutA C792A. The two protomers are colored gray and slate. The 9 proline molecules bound to the dimer are shown in spheres.

As in wild-type BjPutA and other PutAs, the two active sites of the BjPutA C792A variant are separated by 42 Å and connected by a tunnel (Figure 2B). The tunnel supports substrate channeling, a feature of the kinetic mechanism of all PutAs studied to date.

The asymmetric unit of the *C*2 crystal form contains a BjPutA dimer (Figure 2C). The dimer is a domain-swapped assembly in which the oligomerization domain of one protomer engages the GSALDH module of the other protomer. An important aspect of dimerization is that the oligomerization domain covers the substrate-channeling tunnel, thus preventing leakage of the intermediate into the bulk medium. We note that BjPutA forms a dimer-of-dimers tetrameter in crystallo and in solution [6]; however, the dimer is the core functional unit of BjPutA, and the tetramer is not essential for catalytic activity or substrate channeling [7].

### 2.2. FAD Conformation

**Figure 3:**
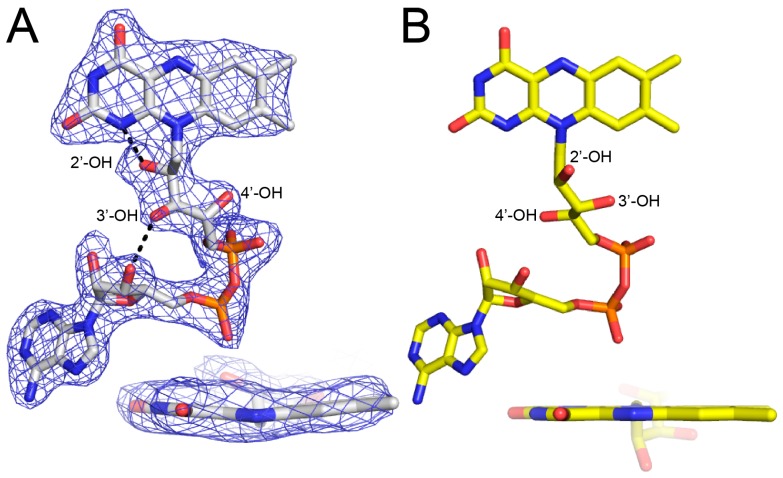
Electron density evidence showing the FAD of BjPutA C792A is reduced. (**A**) The FAD of proline-soaked BjPutA C792A. The mesh represents a simulated annealing *F*_o_–*F*_c_ omit map contoured at 3.0σ. The inset shows an edge-on view of the isoalloxazine. (**B**) The FAD of oxidized BjPutA (PDB ID 3HAZ).

The FAD bears the structural hallmarks of 2-electron reduction, consistent with the bleaching of the yellow color of the crystals upon soaking with proline. Electron density maps clearly show the conformation of the ribityl chain and isoalloxazine ring system (Figure 3A). The 2′-OH and 3′-OH groups are below the pyrimidine ring of the isoalloxazine, and the 4′-OH sits under the dimethylbenzene ring. In this conformation, the 2′-OH forms a hydrogen bond with the FAD N1, while the 3′-OH hydrogen bonds with the FAD ribose. This particular ribityl conformation has been observed in the reduced FADs of other PutAs and is diagnostic of the 2-electron reduced state [7,8,9]. Note that the ribityl conformation of the oxidized FAD in BjPutA is substantially different (Figure 3B).

The isoalloxazine conformation is also consistent with reduction of the FAD in crystallo. The isoalloxazine ring system is planar in oxidized PutAs and monofunctional PRODHs (Figure 3B), whereas it exhibits butterfly bending (*si* face convex) in the reduced enzymes [7,8,9,10,11,12]. The isoalloxazine of BjPutA C792A exhibits a butterfly deformation of 9–13° from planar (*si* face convex) (Figure 3A). We note the butterfly angles in other reduced PutAs are larger, spanning the range of 23–35°. Although the degree of bending is lower in BjPutA C792A, the direction of bending is the same as in other reduced PutAs. These results confirm the FAD in the BjPutA C792A structure is reduced.

### 2.3. Proline Binding Sites

**Figure 4:**
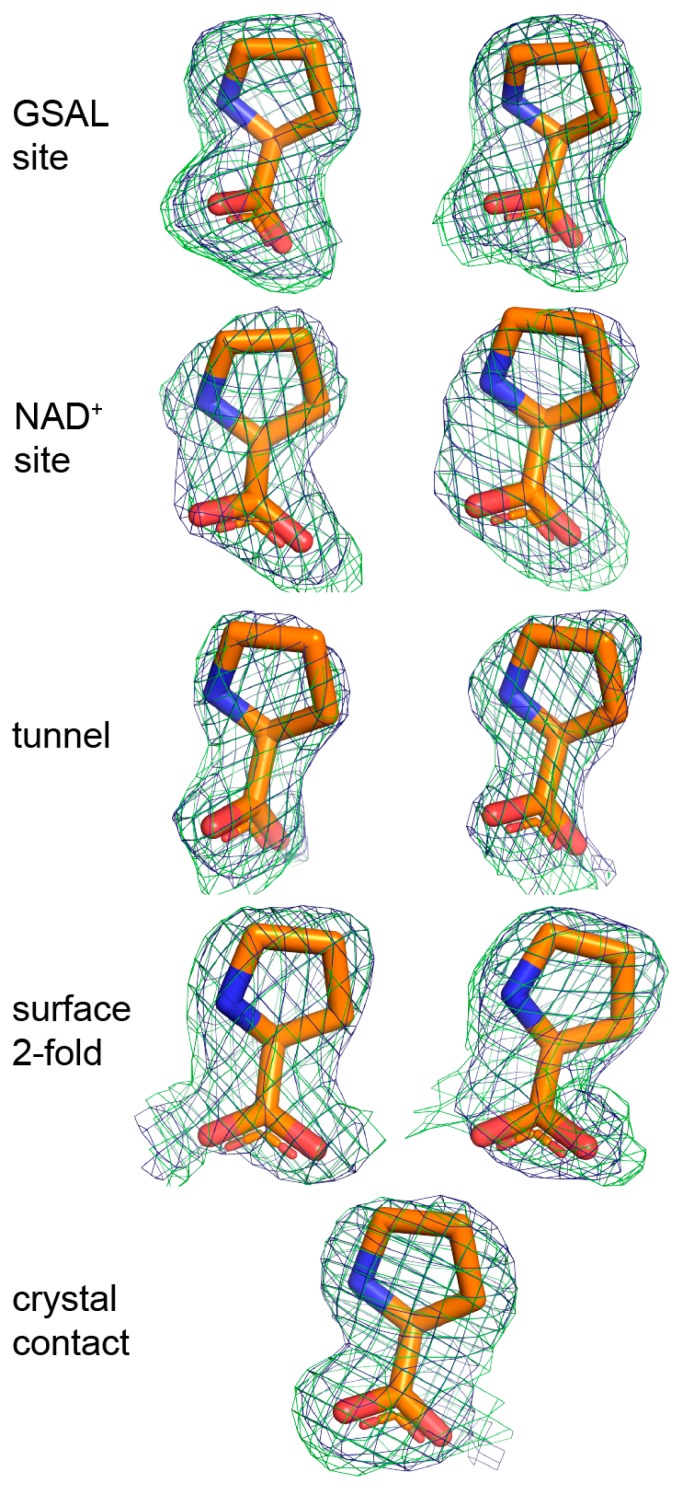
Electron density of the 9 proline molecules bound to C792A. The green mesh represents a simulated annealing *F*_o_–*F*_c_ omit map contoured at 2.5σ. The blue mesh represents the refined 2*F*_o_–*F*_c_ map calculated from the final model, including proline ligands (1.0σ). The left and right sides of the figure show prolines bound to Chains A and B, respectively. Proline in the crystal contact does not have a non-crystallographic symmetry mate.

The electron density maps suggested that several molecules of proline were bound to the enzyme (Figure 4). Nine proline molecules were modeled into 5 sites (Figure 2C, Table 2). Three of the sites are functionally significant (Figure 2B): (1) the GSAL binding site of the GSALDH module; (2) the NAD^+^ site; and (3) the substrate-channeling tunnel. Proline was also modeled into density on the surface of the protein near the 2-fold axis of the dimer and a site stabilized by crystal contacts (Figure 2C); these surface prolines likely represent adventitious binding caused by the high concentration of proline used in crystal soaking (2 M). We note that adventitious binding has been observed in several cases where proline has been used for cryoprotection [13].

Considering the three potentially significant sites, proline appears to be most tightly bound to the GSAL site. For example, the strongest electron density for proline was found in the GSAL binding (Figure 4). Accordingly, proline in the GSAL site has the highest occupancy (1.0) and lowest B-factor of all the sites (Table 2). Note that the mean B-factor of proline in the GSAL site (36 Å^2^) is similar to that of the FAD (33 Å^2^) and lower than that of the protein (40 Å^2^). Further, proline in the GSAL site is stabilized by four direct hydrogen bonds to the protein, whereas proline in the NAD^+^ site forms only one direct hydrogen bond, and proline in the tunnel lacks direct hydrogen bonds with the protein (Table 2). Thus, although the prolines in the NAD^+^ site and the middle of the tunnel occupy functionally relevant locations, their lack of significant hydrogen bonding with the protein suggest that they may not play a major role in substrate inhibition of the coupled reaction.

Interestingly, the maps did not indicate proline bound in the proline pocket of the PRODH active site. Instead, the density suggested the presence of a sulfate ion having modest occupancy (0.8–0.9) and a high B-factor (~95 Å^2^). We note that the structure of wild-type BjPutA also contains a sulfate ion in this location (PDB ID 3HAZ).

### 2.4. Proline Bound in the GSAL Site

**Figure 5:**
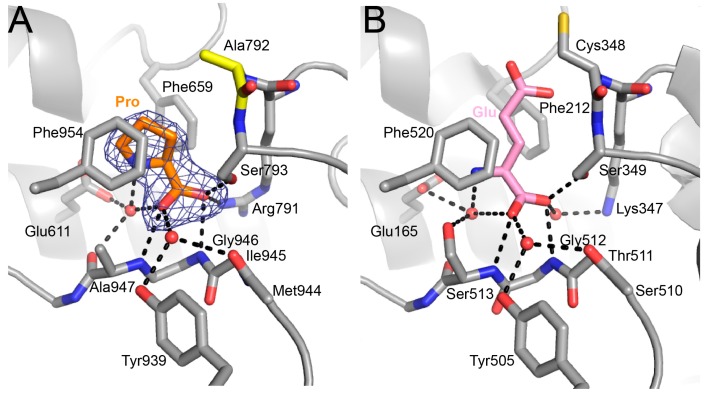
Proline inhibits the GSAL site of the GSALDH module. (**A**) Electron density and interactions for proline bound in the GSAL site. The mesh represents a simulated annealing *F*_o_–*F*_c_ omit map contoured at 3.0σ. (**B**) The active site of mouse GSALDH (ALDH4A1) complexed with the product glutamate (PDB ID 3V9K). Water molecules that mediate enzyme-ligand hydrogen bonds are represented by red spheres.

Proline in the GSAL site forms several interactions with the enzyme (Figure 5A). The carboxylate group interacts with Arg791, Ser793, and the backbone amine groups of Gly946 and Ala947. The amino group of proline forms a water-mediated hydrogen bond with Glu611. The ring of proline is flanked by two Phe residues, Phe659 and Phe954.

Proline in the GSALDH module mimics the substrate GSAL. The recognition of GSAL has been characterized from crystal structures of monofunctional GSALDH (a.k.a. ALDH4A1) complexed with the product glutamate [14,15]. Figure 5 compares the proline site identified here with the structure of mouse GSALDH complexed with glutamate. In both structures, the α-carboxylate hydrogen bonds with the backbone amine groups of a Gly-containing active site loop. This loop, known as the “aldehyde anchor loop,” is a conserved element of substrate recognition in GSALDHs and the related enzyme, α-aminoadipate semialdehyde dehydrogenase (a.k.a. ALDH7A1) [16,17]. Additionally, in both structures, the α-carboxylate of the ligand interacts with a positively charged residue that is immediately *N*-terminal to the catalytic Cys (Arg791 in BjPutA; Lys347 in ALDH4A1). Moreover, both ligands form a water-mediated interaction with a conserved Glu (Glu611 in BjPutA; Glu165 in ALDH4A1). Finally, both ligands are flanked by Phe side chains. These residues form a conserved ALDH substrate recognition motif known as the “aromatic box” [18]. In summary, proline uses several conserved features of substrate recognition to bind in the GSAL site of BjPutA.

### 2.5. Inhibition of Puta GSALDH Activity by Proline

The structure suggests that proline inhibits the GSALDH activity of PutA. This idea was tested using steady-state kinetic measurements of the BjPutA R456M mutant. Arg456 is located in the PRODH active site and is conserved in PutAs and monofunctional PRODHs. Several structures have shown that this residue binds the carboxylate of the substrate proline [5,8,12,19,20,21]. Previous studies showed that BjPutA R456M lacks PRODH activity and exhibits wild-type GSALDH activity. Therefore, BjPutA R456M can be used to study proline inhibition of the PutA GSALDH activity without interference from the PRODH activity.

**Figure 6:**
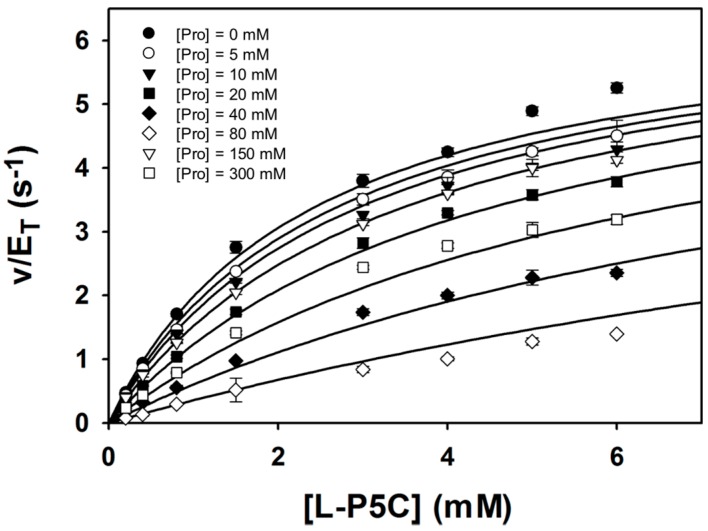
Kinetic data showing that proline is a competitive inhibitor of the GSALDH activity of BjPutA. Initial velocity pattern for BjPutA mutant R456M (0.25 μM) as a function of P5C concentration at 8 different fixed proline concentrations. The curves represent a global fit of the data to a competitive inhibition model. Best fit parameters were *K*_m_ = 2.4 ± 0.1 mM, *k*_cat_ = 6.7 ± 0.1 s^−1^, and *K*_i_ = 46.3 ± 1.7 mM proline.

Steady-state kinetic data were obtained with P5C/GSAL as the varying substrate at several fixed concentrations of proline (Figure 6). The data could be satisfactorily fit to a competitive inhibition model, yielding kinetic constants of *K*_m_ = 2.4 ± 0.1 mM, *k*_cat_ = 6.7 ± 0.1 s^−1^, and *K*_i_ for proline of 46.3 ± 1.7 mM. These results suggest that proline inhibits the GSALDH reaction by binding in the GSAL site, which is consistent with the crystal structure. Furthermore, the *K*_i_ obtained here for proline inhibiting the GSALDH activity of BjPutA (46 mM) is similar to the *K*_i_ for proline inhibiting the coupled reaction obtained previously (24 mM) [3]. Altogether, our results suggest that proline binding in the GSAL site is the structural basis for substrate inhibition of the PutA coupled reaction.

## 3. Materials and Methods 

### 3.1. X-ray Crystallography

The BjPutA mutant C792A was expressed and purified as described [6,22]. Centered monoclinic crystals were grown in sitting drops at room temperature using a reservoir solution containing 2 M ammonium sulfate and 0.1 M Tris at pH 7–8 as described [22]. To form the proline complex, crystals were soaked in the reservoir supplemented with 2 M l-proline. We note that proline provides cryoprotection at this concentration [13]. X-ray diffraction data were collected at the Advanced Light Source beamline 4.2.2 using a NOIR-1 CCD detector. The space group is *C*2 with unit cell parameters of a = 166.8 Å, b = 194.2 Å, c = 108.7 Å, and β = 121.4°. The asymmetric unit contains a BjPutA dimer, and the solvent content based on the methods of Matthews [23] is 65% (*V*_m_ = 3.5 Å^3^/Da). The data were integrated and scaled with XDS [24]. AIMLESS was used to merge reflections and convert intensities to amplitudes. Table 1 lists the data processing statistics. 

Structure refinement in PHENIX [25,26] was initiated from the coordinates of wild-type BjPutA (PDB ID 3HAZ) [6]. Non-crystallographic symmetry restraints were applied throughout refinement. The B-factor model consisted of TLS refinement (one group per protein chain) plus an isotropic B-factor for each non-hydrogen atom. COOT was used for model building [27]. Model validation was carried out using MOLPROBITY [28]. The final model includes 1949 out of the expected 2002 amino acid residues in the asymmetric unit. Each BjPutA protomer contains a reduced FAD (occupancy = 1; PDB ligand code FDA). Two residues were modeled with dual side chain conformations (Arg146 and Ser214). The solvent model includes 719 water molecules (occupancy = 1) and 14 sulfate ions (occupancy = 0.67–0.93). Table 1 lists the refinement statistics.

### 3.2. Steady-State Kinetics Measurements

The BjPutA R456M mutant was expressed and purified as described [3,6]. DL-P5C was synthesized as described and stored in 1 M HCl at 4 °C [29]. P5C was quantified using *o*-aminobenzaldehyde and neutralized to pH 7.5 with 10 M NaOH immediately prior to assays as previously described [4,29,30]. The concentration of l-P5C is considered to be half the total DL-P5C concentration. Inhibition of GSALDH activity by proline was examined by varying l-P5C (0.2–6 mM) at different fixed concentrations of l-proline (0 mM, 5 mM, 10 mM, 20 mM, 40 mM, 80 mM, 150 mM, and 300 mM). Assays were performed as described at 23°C in 50 mM potassium phosphate (pH 7.5, 600 mM NaCl) with 0.25 μM BjPutA R456M mutant and 200 μM NAD^+^ [3]. GSALDH activity was measured by monitoring the formation of NADH at 340 nm (ε = 6200 cm^−1^ M^−1^). Initial velocity data were fitted globally using SigmaPlot 12 to the nonlinear form of the competitive inhibition equation (Equation (1)), where [S] is the varied P5C concentration, *K*_m_ is the Michaelis–Menten constant for P5C, [I] is the proline concentration, and *K*_i_ is the competitive inhibition constant for proline.

(1) $$\frac{v}{{\left[E\right]}_{T}}\;=\frac{k_{\mathrm{cat}}\left[S\right]}{K_{m}\left(1+\frac{\left[I\right]}{K_{i}}\right)+\left[S\right]}.$$

## 4. Conclusions

Substrate inhibition of the coupled PRODH-GSALDH reaction by proline is a hallmark of PutAs. Inhibition of PutA by proline may be advantageous during osmotic stress, when bacteria need to accumulate high levels of proline rather than catabolizing it. The inhibition of the PutA GSALDH site by proline would also lead to a build-up of P5C, which is a competitive inhibitor of the PutA PRODH activity [4]. Thus, under osmotic stress, the substrate inhibition of PutA by proline provides a mechanism for downregulating proline catabolism in favor of accumulating proline. Our results suggest this phenomenon is due to inhibition of the GSALDH reaction caused by proline binding in the GSAL site. The similarity of the interactions formed by proline compared to those of the true substrate GSAL suggests the occupancy of proline in this site is specific, not adventitious. Furthermore, early studies showed that proline is a completive inhibitor (with GSAL) of monofunctional GSALDH (a.k.a. ALDH4A1) [31], and the structure of a monofunctional GSALDH complexed with proline [13] is very similar to the PutA–proline complex described here. We conclude that proline binding in the GSAL site is the structural basis for the substrate inhibition of PutA.

## Figures and Tables

**Table 1:** Data collection and refinement statistics.

Space Group	*C*2
Unit cell parameters (Å, °)	*a* = 166.8 *b* = 194.2 *c* = 108.7 *β* = 121.4
Wavelength	1.000
Resolution (Å)	47.02–2.15 (2.19–2.15)^a^
Observations	587,377 (21,732)
Unique reflections	158,884 (7,418)
*R*_merge_(*I)*	0.089 (0.787)
*R*_meas_(*I*)	0.105 (0.975)
*R*_pim_(*I*)	0.054 (0.563)
Mean *I/*σ	8.7 (1.7)
Mean *CC*_1/2_	0.996 (0.649)
Completeness (%)	99.5 (94.1)
Multiplicity	3.7 (2.9)
No. protein residues	1949
No. of atoms	
Protein	14540
FAD	106
Proline	72
Sulfate ions	70
Water	719
*R*_work_	0.209 (0.320)
*R*_free_^b^	0.239 (0.379)
RMSD bond lengths (Å)	0.007
RMSD bond angles (°)	0.890
Ramachandran plot ^c^	
Favored (%)	98.39
Outliers (%)	0.05
Clashscore (PR) ^c^	2.69 (99%)
MolProbity score (PR) ^c^	1.31 (99%)
Average B-factor (Å^2^)	
Protein	39.6
FAD	32.9
Proline	48.1
Sulfate ions	79.4
Water	37.3
Coordinate error (Å) ^d^	0.28
PDB ID	6BSN

^a^ Values for the outer resolution shell of data are given in parentheses. ^b^ 5% test set. ^c^ From MolProbity. The percentile ranks (PR) for Clashscore and MolProbity score are given in parentheses. ^d^ Maximum likelihood-based coordinate error estimate reported by phenix.refine.

**Table 2:** Refined B-factors and occupancies of proline ligands.

Site	*B*-Factor (Å^2^)	Occupancy	Hydrogen Bonds to the Protein
GSAL site	35.8	1.00	4
GSAL site	35.3	1.00	4
NAD^+^ site	44.1	0.89	1
NAD^+^ site	55.8	0.95	1
Middle of the tunnel	54.6	0.89	0
Middle of the tunnel	61.5	0.90	0
Surface, near the dimer 2-fold axis	47.6	0.89	3
Surface, near the dimer 2-fold axis	49.8	0.92	3
Crystal contact	48.1	0.95	1
